# Early initiation of fast‐track care for persons living with HIV initiating dolutegravir‐based regimens during a period of severe civil unrest in Port‐au‐Prince, Haiti: a pilot randomized trial

**DOI:** 10.1002/jia2.26419

**Published:** 2025-02-09

**Authors:** Jean Bernard Marc, Samuel Pierre, Othnia Ducatel, Fabienne Homeus, Abigail Zion, Vanessa R. Rivera, Nancy Dorvil, Patrice Severe, Colette Guiteau, Vanessa Rouzier, Ingrid T. Katz, Carl Frederic Duchatelier, Guyrlaine Pierre Louis Forestal, Josette Jean, Guirlaine Bernadin, Emelyne Droit Dumont, Rose Cardelle B. Riche, Jean William Pape, Serena P. Koenig

**Affiliations:** ^1^ Haitian Group for the Study of Kaposi's Sarcoma and Opportunistic Infections (GHESKIO) Port‐au‐Prince Haiti; ^2^ Analysis Group Boston Massachusetts USA; ^3^ Weill Cornell Medicine New York New York USA; ^4^ Brigham and Women's Hospital, Harvard Medical School Boston Massachusetts USA

**Keywords:** antiretroviral therapy, differentiated service delivery, HIV, rapid care, resource‐poor setting, treatment

## Abstract

**Introduction:**

Differentiated service delivery (DSD) models have been widely implemented for patients in stable HIV care. However, DSD has rarely been offered to newly diagnosed patients. We assessed the effectiveness of early fast‐track care during a period of severe civil unrest in Port‐au‐Prince, Haiti.

**Methods:**

We conducted a pilot randomized trial among adults presenting with early HIV disease to determine whether early fast‐track care (8−12 weeks after same‐day HIV testing and antiretroviral therapy [ART] initiation) was associated with superior outcomes compared with standard (deferred eligibility for fast‐track care). All participants received tenofovir/lamivudine/dolutegravir (TLD), and HIV‐1 RNA <200 copies/ml was required prior to initiating fast‐track care. The primary outcome was 48‐week HIV‐1 RNA <200 copies/ml, with intention‐to‐treat analysis.

**Results:**

From December 2020 to August 2022, 245 participants were randomized to standard (*n* = 116) and early fast‐track (*n* = 129) groups. All initiated TLD on the day of HIV diagnosis. In the early fast‐track group, one (0.8%) died, 12 (9.3%) were internally displaced/emigrated, five (3.9%) were lost‐to‐follow‐up (LTFU), two (1.6%) had a gap in care/later return, one (0.8%) was transferred and 108 (83.7%) were retained; 88 (68.2%) received 48‐week viral load testing and 80 (90.9% of tested; 62.0% of randomized) had HIV‐1 RNA <200 copies/ml. In the standard group, two (1.7%) died, six (5.2%) were internally displaced/emigrated, three (2.6%) were LTFU, one (0.9%) had a gap in care/later return, one (0.9%) was transferred and 103 (88.8%) were retained; 78 (67.2%) received 48‐week viral load testing and 66 (84.6% of tested; 56.9% of randomized) had HIV‐1 RNA <200 copies/ml. By design, the sample size of this pilot study was too small to provide definitive evidence of treatment effect, but the primary outcome was numerically higher in the early fast‐track group (62.0% vs. 56.9%; RD: 0.051: 95% CI: −0.072, 0.174).

**Conclusions:**

Early fast‐track care was associated with high levels of viral suppression among adults initiating same‐day TLD, despite severe civil unrest in Haiti. Completion of 48‐week viral load testing was suboptimal, due to the need for participants to leave Port‐au‐Prince during peak periods of gang‐related violence, and the lack of availability of viral load testing for those receiving non‐facility‐based ART.

## 1 INTRODUCTION

Differentiated service delivery (DSD) models have been widely implemented to facilitate follow‐up care for persons living with HIV (PLWH). Multiple studies have demonstrated excellent outcomes with DSD strategies, including fewer visits, multi‐month dispensing of medications and decentralized drug distribution [1, 2, 3, 4, 5, 6, 7, 8, 9, 10]. However, expedited services are generally offered only to PLWH considered to be stable after at least six months of antiretroviral therapy (ART) [1, 7, 8]. Data on outcomes of earlier DSD eligibility are limited [11]. With the widespread use of dolutegravir (DTG)‐based ART regimens in low‐ and middle‐income countries, viral suppression can be achieved within 8−12 weeks [12]. This provides the potential opportunity for an earlier transition to expedited services, during the period when loss‐to‐follow‐up (LTFU) rates are the highest.

We conducted a pilot randomized trial to determine whether early fast‐track care (at 8−12 weeks after ART initiation) was associated with superior outcomes compared with standard care (deferred eligibility for fast‐track care) in Haiti. We hypothesized that early fast‐track care would improve the primary outcome of retention with viral suppression at 48 weeks after enrolment.

## 2 METHODS

### 2.1 Study design and setting

We conducted a pilot randomized trial to compare rates of viral suppression with early fast‐track versus standard care among patients newly diagnosed with HIV at the Haitian Group for the Study of Kaposi's Sarcoma and Opportunistic Infections (GHESKIO), a Haitian non‐governmental organization that provides care for infectious and non‐communicable diseases in Port‐au‐Prince, Haiti. The adult HIV prevalence in Haiti is an estimated 1.7% [13]. This study was conducted during a period of severe political instability in Haiti [14]. Armed gangs control over 80% of Port‐au‐Prince, with widespread violence that has resulted in the closure of 80% of clinics and hospitals, and the internal displacement of over 700,000 people [15, 16, 17].

### 2.2 Ethics statement

The study was approved by the institutional review boards at GHESKIO, Brigham and Women's Hospital and Weill Cornell Medical College. Written informed consent was obtained from all participants.

### 2.3 Study participants

Patients newly diagnosed with HIV were recruited for this study at the GHESKIO outpatient clinic. Key inclusion criteria included ≥18 years of age and WHO Stage 1 or 2 disease; exclusion criteria included previous ART, pregnancy, creatinine clearance (CrCl) <50 or alanine transaminase (ALT) or aspartate transaminase (AST) >5 times the upper limit of normal.

### 2.4 Randomization and masking

Participants were randomized in a 1:1 ratio using a computer‐generated random‐number list in the GHESKIO Data Management Unit by a data manager who had no other involvement in study procedures. Study staff were not blinded to the randomization group.

### 2.5 Study procedures

All participants initiated tenofovir/lamivudine/dolutegravir (TLD) on the day of HIV diagnosis and were enrolled and randomized within the subsequent three days. Specimens were collected for creatinine, ALT, AST, complete blood count and CD4 count. Same‐day lab results were not available, so participants meeting exclusion criteria were late exclusions. For both groups, eligibility for fast‐track care required being on time for the clinic visit (≤3 days late), without an active WHO Stage 3 or 4 condition and with HIV‐1 RNA <200 copies/ml. The only difference between the two groups was the timing of eligibility for fast‐track care.

In the standard group, the visit schedule initially included monthly visits for the first six months, and then quarterly visits. However, in mid‐2021, the standard of care changed to quarterly visits. At the 24‐week visit, participants meeting eligibility criteria initiated fast‐track care. In the early fast‐track group, participants received the same care as the standard group for the first eight weeks. At the week 8 visit, viral load testing was conducted, and participants who met eligibility criteria initiated fast‐track care; those who were not yet eligible were re‐assessed at the week 12 visit, and if not eligible, they were re‐evaluated at subsequent visits.

The fast‐track care intervention was based on the “when, where, who, and what” components of the DSD model [18]. Fast‐track care included quarterly visits and ART prescriptions; however, some participants received longer durations of ART if necessary for travel outside of Port‐au‐Prince. Participants chose the location of their visits, which took place either at the GHESKIO facility or one of nine community ART sites, which have been described previously [19]; for those facing severe challenges in attending visits, home delivery of ART was offered. Viral load testing capacity was being scaled up at community sites during the study period, and not available with home ART delivery. Visits were conducted by a study nurse. At each visit, pre‐packaged ART and other standard medications were dispensed at the point‐of‐care. Fast‐track visits were expedited, with minimal waiting time. To facilitate retention, participants received phone calls prior to visits and after missed visits, a transportation subsidy of 100 Haitian gourdes (about $US 1.00) and a phone card (valued at 100 Haitian gourdes) at each visit. Participants with HIV‐1 RNA ≥200 copies/ml received adherence counselling, followed by repeat viral load testing.

### 2.6 Outcomes

The primary outcome was the proportion of participants with a 48‐week viral load <200 copies/ml, with a prespecified window of 47−60 weeks; missing test results were considered virologic failures. Secondary outcomes included: 48‐week HIV‐1 RNA cut‐offs of <50 and <1000 copies/ml; adherence as measured by the proportion of days covered (proportion of days ART was available within the study period); and tolerability, as measured by permanently discontinuing TLD due to adverse events. Participants were considered retained in care at 48 weeks if they attended a visit from 36 to 60 weeks after enrolment.

### 2.7 Statistical analysis

For the primary outcome, a sample size of 242 was calculated to provide 80% power to detect an absolute difference of 11% in the proportion of participants with 48‐week HIV‐1 RNA <200 copies/ml (standard: 70%; early fast‐track: 81%) assuming a significance level of 0.25, using the Chi‐square test. We used the method of Schoenfeld, in which preliminary hypothesis testing for efficacy is conducted with a high type I error rate with the goal of informing the design of a larger randomized trial to follow, and not to provide definitive evidence of treatment effect [20, 21, 22]. The sample size was inflated to 247 to account for late exclusions.

We compared the proportion of participants with 48‐week HIV‐1 RNA <200 copies/ml (primary endpoint) and binary secondary outcomes using the Chi‐square test (Fisher's exact test was used for sparse data). We presented unadjusted risk difference (RD) with a 95% confidence interval (CI) using the Wilson score interval. Results were disaggregated by sex for the primary outcome. We also conducted multivariable regression to control for any unbalanced baseline characteristics. All analyses were conducted using SAS version 9.4. The study was registered with ClinicalTrials.gov NCT04311944.

## 3 RESULTS

**Figure 1:**
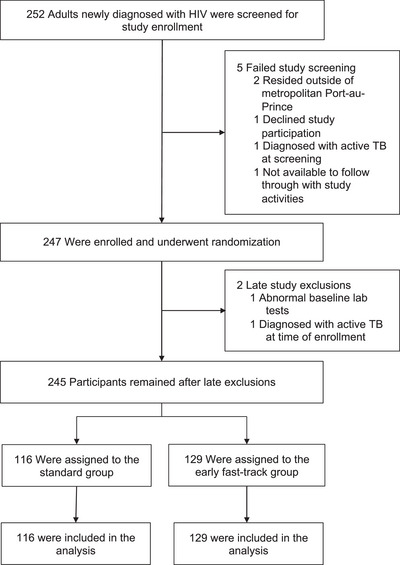
CONSORT diagram: screening, randomization, and analysis populations.

From 13 December 2020, to 19 August 2022, 252 patients were screened and 247 (98.0%) were enrolled and randomized (Figure 1). Two participants met pre‐specified criteria for late exclusions, leaving 245 included in the analyses (standard: 116; early fast‐track: 129). The final study visit occurred on 6th September 2023.

Baseline characteristics are described in Table 1. The median time from HIV diagnosis to enrolment was 0 days (interquartile range [IQR]: 0, 0). Among the 116 participants in the standard group, 81 (69.8%) received 24‐week viral load testing, and 73 (90.1% of tested; 62.9% of randomized) had 24‐week <200 copies/ml and were eligible for fast‐track care. Among the 129 participants in the early fast‐track group, 100 (77.5%) received 8‐week viral load testing and 97 (97.0% of tested; 75.2% of randomized) had HIV‐1 RNA <200 copies/ml. Of the remaining 32 participants in the early fast‐track group, 15 received a 12‐week viral load, and 11 (73.3%) had HIV‐1 RNA <200 copies/ml. In total, 108 (83.7%) of participants in the intervention group initiated fast‐track care within 12 weeks after enrolment.

**Table 1:** Baseline characteristics of study participants by group

	Standard care group (*n* = 116)	Early fast‐track group (*n* = 129)
Female—no. (%)	46 (39.7)	64 (49.6)
Age at enrolment (years)—median (IQR)	40 (36, 47)	40 (33, 48)
Income—no. (%)		
<$US 1.00 per day	82 (70.7)	93 (72.1)
≥$US 1.00 per day	14 (12.1)	10 (7.8)
Missing	20 (17.2)	26 (20.2)
Education level—no. (%)		
None	16 (13.8)	11 (8.5)
Preschool or some primary	28 (24.1)	38 (29.5)
At least some secondary	52 (44.8)	54 (41.9)
Missing	20 (17.2)	26 (20.2)
Marital status—no. (%)		
Single	29 (25.0)	30 (23.3)
Married	46 (39.7)	42 (32.6)
Formerly married	21 (18.1)	31 (24.0)
Missing	20 (17.2)	26 (20.2)
CD4 count (cells/mm^3^)—median (IQR)	293 (154, 451)	318 (191, 485)
CD4 count category—no. (%)		
<100 cells/mm^3^	14 (12.1)	9 (7.0)
100–349 cells/mm^3^	43 (37.1)	39 (30.2)
350–499 cells/mm^3^	17 (14.7)	15 (11.6)
≥500 cells/mm^3^	19 (16.4)	19 (14.7)
Missing	23 (19.8)	47 (36.4)

Abbreviation: IQR, interquartile range.

Among the 129 participants in the early fast‐track group, 108 (83.7%) were retained and 88 (68.2%) received 48‐week viral load testing. Eighty (90.9% of tested; 62.0% of randomized) had HIV‐1 RNA <200 copies/ml. Among the 116 participants in the standard group, 103 (88.8%) were retained, and 78 (67.2%) participants received 48‐week viral load testing (Table 2). Sixty‐six participants (84.6% of tested; 56.9% of randomized) had HIV‐1 RNA <200 copies/ml. The proportion of participants achieving the primary outcome was numerically higher in the early fast‐track group (62.0% vs. 56.9%; RD: 0.051: 95% CI: −0.072, 0.174), though the difference was not statistically significant. Results were similar in the adjusted analysis (RD: 0.069; 95% CI: −0.080, 0.218). There was no difference in the primary outcome when stratified by sex. In secondary analyses, results were similar with HIV‐1 RNA cut‐offs of <50 copies and <1000 copies/ml (Table 2).

**Table 2:** Primary and secondary outcomes by group

Outcome	Standard care group (*n* = 116)	Early fast‐track group (*n* = 129)	Risk difference (95% CI)
**Primary outcome**			
48‐week HIV‐1 RNA <200 copies/ml	66 (56.9%)	80 (62.0%)	0.051 (−0.072, 0.174)
Received 48‐week viral load testing	78 (67.2%)	88 (68.2%)	0.010 (−0.108, 0.127)
HIV‐1 RNA <200 copies/ml among those receiving viral load testing	66/78 (84.6%)	80/88 (90.9%)	0.063 (−0.037, 0.163)
**Secondary outcomes**			
48‐week HIV‐1 RNA <50 copies/ml	61 (52.6%)	75 (58.1%)	0.056 (−0.069, 0.180)
48‐week HIV‐1 RNA <1000 copies/ml	70 (60.3%)	83 (64.3%)	0.040 (−0.082, 0.161)
*Forty‐eight week outcomes* ^a^			
Retained in care at 48 weeks	103 (88.8%)	108 (83.7%)	−0.051 (−0.136, 0.035)
Died	2 (1.7%)	1 (0.8%)	−0.009 (−0.038, 0.019)
Internally displaced or emigrated	6 (5.2%)	12 (9.3%)	0.013 (−0.031, 0.057)
Lost‐to‐follow‐up	3 (2.6%)	5 (3.9%)	0.041 (−0.023, 0.106)
Gap in care—returned after 48‐week visit window	1 (0.9%)	2 (1.6%)	0.007 (−0.020, 0.034)
Transferred	1 (0.9%)	1 (0.8%)	−0.001 (−0.023, 0.022)

^a^ These are mutually exclusive outcomes which include all 245 participants.

In the early fast‐track group, one (0.8%) died, 12 (9.3%) were internally displaced to the provinces or emigrated, five (3.9%) were LTFU, two had a gap in care with later return and one (0.9%) was transferred (Table 2). In the standard group, two participants (1.7%) died, six (5.2%) were internally displaced or emigrated, three (2.6%) were LTFU, one (0.9%) had a gap in care with later return and one (0.9%) was transferred. Median adherence was 98.8% (IQR 89.6%, 100.0%) in the early fast‐track and 96.9% (IQR: 90.1%, 100%) in the standard group. No participant stopped TLD due to adverse events.

## 4 DISCUSSION

This study was conducted during a period of severe civil unrest and gang violence in Haiti, which presented extraordinary challenges to the health system. We found that the proportion of participants with virologic suppression was numerically higher with early fast‐track care, compared to standard care, though by design, the sample size of this proof‐of‐concept study was insufficiently powered to demonstrate definitive evidence of treatment effect. A definitive trial is not planned, because the early fast‐track intervention is now being implemented at GHESKIO. Earlier access to expedited visits and choice of visit location facilitates care in the challenging circumstances in Port‐au‐Prince.

Our study is novel in that the intervention group was eligible for fast‐track care as early as eight weeks after ART initiation. In Rwanda, a pilot study found that early access to expedited facility‐based DSD services (at six months after ART initiation) after one or two suppressed viral loads was feasible, acceptable to patients and associated with similar rates of viral suppression as standard care [11]. It is noteworthy that all participants in our study initiated ART on the day of HIV diagnosis. Despite the circumstances in Port‐au‐Prince, rates of retention and viral suppression were equal or superior to the intervention groups in other same‐day ART studies [11, 23, 24, 25, 26, 27, 28]. We attribute this to the high potency and tolerability of DTG, as well as the streamlined provision of follow‐up care; these interventions have also been incorporated into other successful rapid ART programmes [23, 24, 25, 27, 29, 30, 31].

It is important to emphasize that due to gang‐related violence, the GHESKIO staff needed to employ a variety of strategies to provide access to ART, particularly during peak periods of violence. For this reason, 48‐week viral load testing within the window period of 47−60 weeks was not feasible for all participants who remained in care and taking ART. Though viral load testing capacity was always available at GHESKIO, it was not always available at community sites, and generally unavailable for those who required home ART delivery (due to the danger of leaving their neighbourhood), or longer durations of ART for travel from Port‐au‐Prince to the Haitian provinces or the Dominican Republic.

We note that DSD models of care have also been found to be effective in other settings of civil unrest [32, 33, 34]. Medecins Sans Frontiers provides DSD models of care in conflict‐affected areas in South Sudan, Central African Republic (CAR) and Yemen [32]. Similar to our study, these DSD projects include multi‐month prescribing, plans for patients to contact medical staff and community strategies for ART prescribing, which have also been found effective in DSD models of care that have been implemented in politically stable countries [1, 2, 3, 4, 5, 6, 9, 10, 31, 32, 33, 34, 35, 36, 37]. In South Sudan, mobile teams provided HIV care, with community ART groups for patients on stable ART, and at 12 months, 68% remained in care; 52% had viral load testing; and 90% of these had virologic suppression [33]. In CAR, “run‐away” bags containing three or four months of ART were distributed; three years later, over half of the patients were still in follow‐up [34]. In Yemen, patients received a health card that included a helpline to call in case of drug shortages, with quarterly visits after the first year in care; at the end of the observation period, 72% were in care [34]. As in our study, internal displacement and emigration were common reasons for loss from care.

Our study was conducted among ART‐naïve patients in a large urban clinic, which may limit the generalizability of our findings. Moreover, study staff were not blinded to the randomization group. This could have resulted in bias, with differential treatment which could have impacted participant outcomes in each group. Furthermore, the study was limited by the small sample size; the study was insufficiently powered to demonstrate definitive evidence of treatment effect. In addition, there were unbalanced characteristics between the standard and early fast‐track groups, though results were similar after controlling for these differences.

## CONCLUSIONS

In conclusion, in this proof‐of‐concept pilot study which was conducted in a setting of severe civil unrest, rates of viral suppression were numerically higher with early fast‐track, compared with standard care. Completion of 48‐week viral load testing was suboptimal, due to the need for participants to leave Port‐au‐Prince during peak periods of gang‐related violence, and the lack of availability of viral load testing for those receiving non‐facility‐based ART.

## COMPETING INTERESTS

All authors report no competing interests.

## AUTHORS’ CONTRIBUTIONS

All authors were involved in drafting the article, and all read and approved the final version to be published. Study conception and design: SP, PS, CG, JWP and SPK: Acquisition of data: JBM, SP, OD, FH, VRR, ND, CG, VR, CFD, GPLF, JJ, GB, EDD and RCBR; Analysis and interpretation of data: JBM, SP, FH, AZ, VRR, PS, VR, ITK, JWP and SPK.

## FUNDING

This study was funded by a grant from ViiV Healthcare.

## Data Availability

If the study is accepted for publication, we will include a de‐identified data as Supporting Information.
